# Optimizing Surface Properties of AISI 409 Stainless Steel through Duplex Treatment Consisting of Plasma Nitriding and Vanadium-Based Deposition

**DOI:** 10.1021/acsomega.5c00885

**Published:** 2025-07-16

**Authors:** André Felipe Soares Do Monte e Silva, Larissa Solano de Almeida, Leandro Almeida Silva, Luciana Sgarbi Rossino, Maelson Sousa Nunes, Maxwell Santana Libório, Thércio Henrique de Carvalho Costa, Rafael Marinho Bandeira, Rômulo Ribeiro Magalhães de Sousa

**Affiliations:** † Postgraduate Program in Materials Science and Engineering, Federal University of Piauí, Teresina, PI 64049-550, Brazil; ‡ Postgraduate Program in Materials Science, 600424 Federal University of São Carlos, São Carlos, SP 13565-905, Brazil; § Postgraduate Program in Chemistry, Federal University of Piauí, Teresina, PI 64049-550, Brazil; ∥ School of Science and Technology, 28123 Federal University of Rio Grande do Norte, Natal, RN 59078-970 Brazil; ⊥ Postgraduate Program in Mechanical Engineering, 28123 Federal University of Rio Grande do Norte, Natal, RN 59078-970, Brazil; # Department of Physical Chemistry, São Paulo State University, São Carlos, SP 13560-970, Brazil; ∇ Department of Mechanical Engineering, Federal University of Piauí, Teresina, PI 64049-550, Brazil

## Abstract

This study proposes a surface modification methodology for AISI 409 stainless steel by combining cathodic cage plasma nitriding (CCPN) and deposition (CCPD), evaluating the benefits of this duplex treatment over individual treatments. The mechanical strength, tribological behavior, and corrosion resistance of the treated surfaces were investigated in relation to processing parameters and resulting microstructures. Analyses were performed using XRD, SEM, Vickers microhardness, ball-on-disc testing, and corrosion testing in a 3.5% NaCl solution. The duplex treatment at 400 and 450 °C, consisting of CCPN followed by CCPD, promoted significant surface modifications. Nitriding resulted in a thick layer of Fe_3_N, Fe_4_N, and CrN, increasing hardness and wear resistance, with final improvements of 5.7 and 33.5 times, respectively. The subsequent VN deposition enhanced corrosion resistance, shifting the potential from −396 mV to −221 mV, indicating reduced electrochemical activity. These results confirm the treatment’s potential for automotive exhaust systems, requiring lightweight, durable materials in aggressive environments.

## Graphical abstract

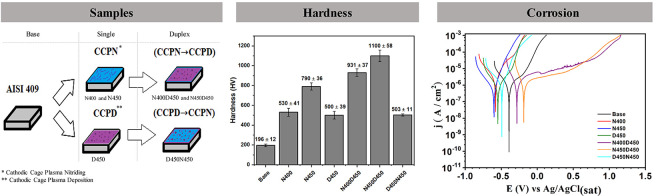

## 1 Introduction

Ferritic stainless steels (FSS), such as AISI 409, combine moderate corrosion resistance and good thermal stability at a relatively low cost, making them attractive for automotive exhaust systems, heat exchangers, and structural components.^1–3^ However, their inherent low hardness and limited wear resistance restrict their application in more demanding environments. Surface engineering via thermochemical treatments has emerged as an effective strategy to overcome these limitations by introducing hard nitride layers while preserving the substrate’s corrosion behavior.^4,5^

Plasma nitriding (PN) is a well-established diffusion process in which nitrogen ions, accelerated by a direct current (DC) bias, bombard the steel surface, promoting nitrogen absorption and the formation of expanded ferrite (αN) or iron nitride (Fe_2–3_N, Fe_4_N) phases.^6,7^ A higher applied bias results in steeper temperature gradients between the surface and the core, enhancing nitrogen diffusion near the surface but potentially inducing subsurface thermal stresses. The electric potential therefore plays a key role in controlling ion energy and heat input, which in turn influence nitride layer thickness, phase composition, and the potential for undesirable CrN precipitation that may compromise corrosion resistance.^8^ Cathodic cage plasma nitriding (CCPN) refines this approach by surrounding the sample with a cathodic cage made of a material in the same category as the sample (e.g., stainless steel), which promotes a more uniform plasma distribution and consistent nitrided layer on complex geometries.^8,9^

Cathodic cage plasma deposition (CCPD), on the other hand, uses a cathodic cage fabricated from a different target material (e.g., vanadium) under identical plasma conditions to deposit a thin nitride film onto the substrate.^10–12^ Parameters such as DC bias and cage geometry affect the film’s thickness and adhesion, while low processing temperatures and controlled gas composition help suppress undesirable chromium nitride formation. Although these parameters have been investigated in isolation, the quantitative relationship between thermal gradients and the coformation of chromium and vanadium nitrides remains scarce, motivating the present study.

Combining CCPN and CCPD in a duplex treatment leverages the complementary benefits of both processes: CCPN forms an interlayer with a hardness gradient that improves adhesion and provides initial wear resistance, while CCPD deposits a harder nitride surface film that enhances protection under more aggressive conditions.^13,14^ Vanadium nitride (VN) coatings have demonstrated excellent mechanical strength and corrosion resistance, making them promising for high-performance applications on both ferrous and nonferrous substrates.^15,16^ Likewise, Cr-based nitrides and composites such as CrN, CrON, and Fe–Cr–V alloys demonstrate high hardness and wear resistance, particularly when processed by plasma-assisted or additive manufacturing techniques.^17,18^ These findings emphasize the role of Cr- and V-based chemistries in advanced surface treatments and justify their incorporation into duplex systems. Recent studies on martensitic and austenitic steels demonstrate that such duplex routes can achieve surface hardness exceeding 1000 HV while maintaining corrosion performance, underscoring the potential of Cr- and V-based chemistries in advanced surface engineering.^19,20^

The present work builds on this foundation, applying CCPN, CCPD, and their duplex combination to AISI 409 ferritic stainless steel. We systematically investigate how processing parameters including electric potential, temperature (400 °C vs 450 °C), and gas ratios (H_2_/N_2_ = 1/3) govern phase formation, microstructure, and the resulting mechanical and corrosion-wear performance. This study aims to provide mechanistic insights into layer formation and to identify an optimized duplex protocol for industrial applications requiring both wear and corrosion protection.

## 2 Materials and Methods

Samples of AISI 409 stainless steel (C = 0.08%, Cr = 11.02%, Mn = 1.00%, Mo = 0.04%, P = 0.05%, S = 0.05%, Ti = 0.48%, balance Fe) were prepared in 15 mm × 15 mm × 5 mm prisms (initial hardness 196 HV). The surface-preparation protocol was adapted from the detailed procedure of previous works,^21^ which originally treated AISI M2 high-speed steel tools (7 × 12 × 19 mm^3^) prior to plasma nitriding. Although the steel grade and sample geometry differ, we retained their multistep grinding and polishing sequence, including 220, 360, 400, 600, and 1200 grit, followed by 3 μm alumina felt polishing, then rinsing in water and 70% ethanol to remove oxides and contaminants. The samples were dried for 10 s using a hot, directional air jet at approximately 50 °C and 150 Pa. This surface preparation protocol ensures surface uniformity and promotes homogeneous nitrogen absorption during CCPN/CCPD treatments, even when substrate composition and dimensions vary.

Plasma treatments were carried out in a reactor connected to a vacuum pump with a base pressure of 0.2 mbar, and a DC source with a maximum voltage of 1200 V, as illustrated in Figure 1 and reported in detail in previous work.^22–24^ The duplex treatment applied to the samples consisted of two stages: plasma nitriding with a cathodic cage of stainless steel (CCPN) and vanadium nitride deposition (VD) using a vanadium cathodic cage. The CCPN and VD underwent a presputtering stage in the same plasma equipment and under floating potential (with the sample on an alumina disk).^24^ This stage involved the exclusive use of inert gases (H_2_/argon = 1) at a temperature of 350 °C and a pressure of 1.3 mbar, lasting for 1 h to remove residual impurities and create void spaces (gaps) on the surface of the samples to facilitate nitrogen diffusion.^25^

**Figure 1:**
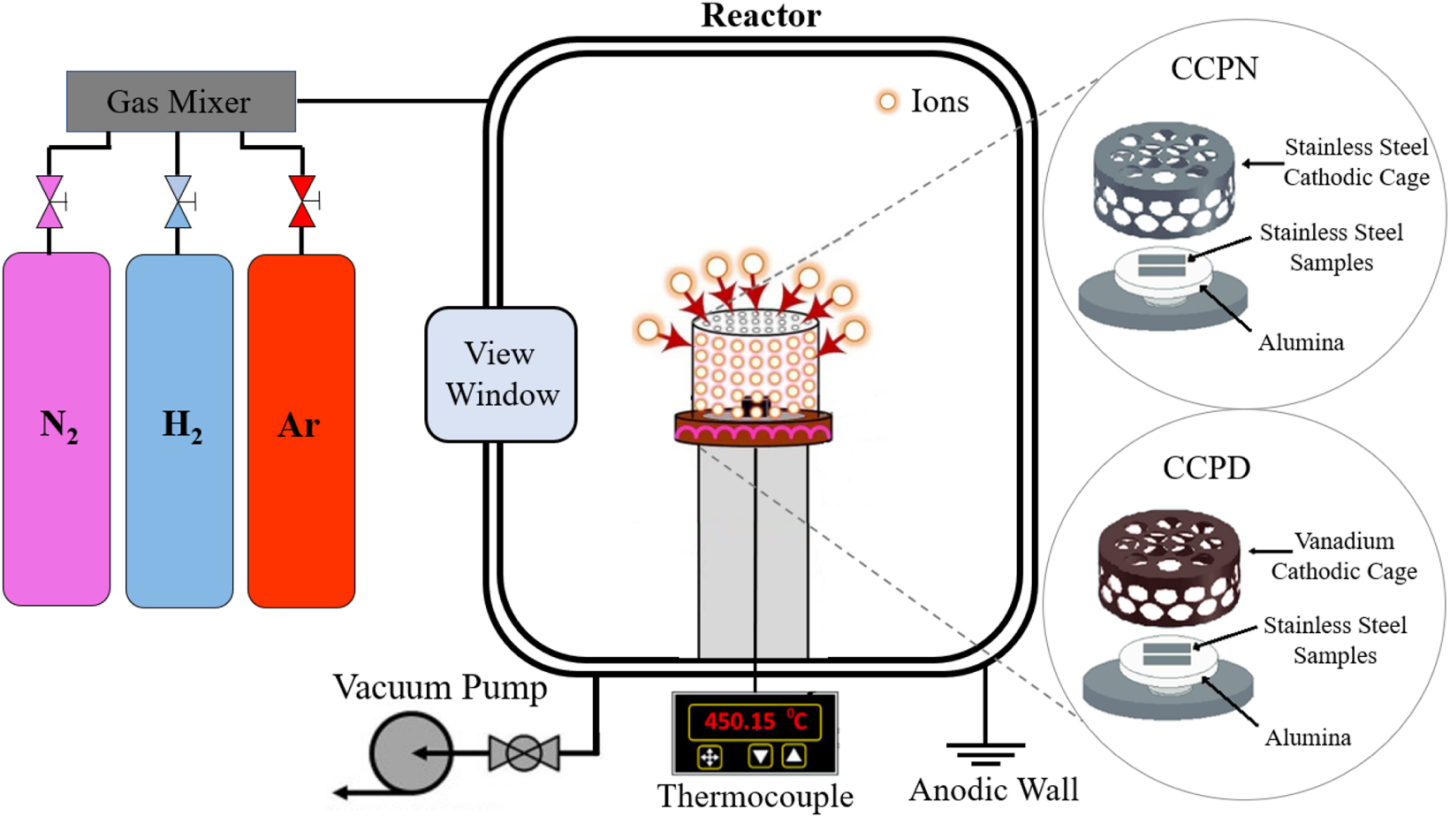
Schematic diagram of CCPN and CCPD highlighting the use of different cathodic cages in each treatment.

All CCPN and VD treatments were carried out with a duration of 4 h at a working pressure of 2.0 mbar under a reactive atmosphere of 25 sccm of H_2_ and 75 sccm of N_2_ (H_2_/N_2_ = 1/3). The H_2_/N_2_ ratio of 1/3 (25% H_2_, 75% N_2_) is commonly adopted because it balances the efficiency of oxide layer removal with the generation of active nitrogen species, resulting in uniform and adherent nitride layers.^26,27^ The temperatures of 400 °C and 450 °C are selected to maximize surface hardness without promoting the formation of undesirable chromium nitrides, thereby preserving good corrosion resistance. These conditions have been optimized in previous studies and successfully applied to ferritic and martensitic stainless steels similar to AISI 409.^28–30^ Each plasma treatment was carried out on three independently prepared AISI 409 samples. The temperature and configuration of the single or duplex treatment shown in Table 1 were the changes adopted for investigation in this work.

**Table 1:** Processing Temperatures in CCPN and VD

Samples	CCPN temperature (°C)	VD temperature (°C)
Base	-	-
N400	400	-
N450	450	-
D450	-	450
N400D450	400	450
N450D450	450	450
D450N450^a^	450	450

a This sample was produced with the order of the treatments reversed (VD followed by CCPN).

The crystalline phases of the coatings were determined by X-ray diffraction (XRD) using a Shimadzu XRD-7000 diffractometer with a Cu–Kα radiation source. The copper target tube was operated at 40 kV and 30 mA. Analyses were performed in −2θ mode over a scanning range of 20° to 80°. Phase identification was carried out using the X́Pert HighScore Plus software. The morphology and thickness of the coatings were evaluated by scanning electron microscopy (SEM), using a Hitachi Tabletop Microscope TM300 with an acceleration voltage of 15 kV for all samples. The Vickers microhardness (HV) test was performed using an Insize microhardness tester (ISH–TDV 1000 A-B) with a load of 50 gf applied for 15 s. For each sample, five measurements were adopted, and the mean value and standard deviation were calculated.^31^

The microabrasive wear tests (MAWTs) were performed using a fixed-ball microwear device. Further information about the device may be found in ref 36. The 52100 steel ball with a 12.7 mm diameter was used. The fixed frequency used in this test was 744 rpm, with a normal load of 8 N at 40 Hz of rotation for 150, 300, 600, 900, and 1200 s. The MAWTs tests were realized in all samples (with and without treatment), and any abrasive or lubricant liquid was used. Four tests were performed on each sample type to improve the accuracy. After tests, all the samples were analyzed using a Leica optical microscope, model DMI8 C, and analysis software, which measured the diameters and the crater radius generated in a coated system by ball rotation.^32^ Therefore, the wear volume and coefficient of friction (COF) were defined by equations better described by Rutherford and Hutchings.^33^ Corrosion measurements were performed in AUTOLAB potentiostat/galvanostat equipment, model PGSTAT302N. Corrosion tests were performed with a three-electrode system in a glass cell using a 3.5% m/m NaCl solution (Dynamics 99%). An Ag/AgCl/KCl­(Sat.) electrode was used as a reference to obtain the potentiodynamic polarization curve. Potentiodynamic polarization measurements started at −900 mV and were interrupted when the current reached 1.3 mA·cm^–2^ at a sweep speed of 1 mV·s^–1^. The open circuit potential (OCP) was measured for 60 s before each potentiodynamic polarization measurement. The linear extrapolation of the cathodic and anodic slope was also obtained from the OCP graphs, in agreement with the methodology presented in previous works.^34,35^

## 3 Results and Discussion

Figure 2 depicts the structural modifications induced by nitriding and cathodic cage deposition processes. Figure 2a shows that plasma nitriding with a cathodic cage at 400 and 450 °C formed a layer composed of iron nitrides and chromium nitrides. It can be observed that the temperature increase (from 400 to 450 °C) results in a reduction of the Fe peaks. This reduction is justified by the increase in the nitrided layer and the addition of the resulting film from the CCPD process with a vanadium cage (Figure 2b).^36^ The presence of nitride precipitates (Fe_3_N, Fe_4_N, and CrN) on the surface of the samples contributes to increasing the mechanical strength of AISI 409, which originally had low hardness.^22^ Additionally, the iron nitrides that constitute the compound layer (the outermost zone of the nitrided layer) can protect the underlying regions when exposed to corrosive environments.^37^ On the other hand, the diffractograms did not show the formation of a solid solution composed of interstitial nitrogen α_N_ in the iron matrix, which would be identified by the shifting of the iron peaks to smaller 2θ angles, but rather the formation of chromium nitride (CrN). Corengia et al. (2004) state that nitriding performed on steels with chromium in their structure can lead to the decomposition of the αN solid solution into α and CrN.^38^ The precipitation of chromium nitride, associated with the reduction of chromium in the solid solution, reduces the ability to form the passivating layer on the surface of the nitrided samples. This can lead to a reduction in corrosion resistance.^22^ The diffractograms also reveal that the Fe_4_N phase appears only in the samples nitrided at 450 °C (2θ equal to 41.3° and 47.9°). This contributes positively to the mechanical resistance of the samples.

**Figure 2:**
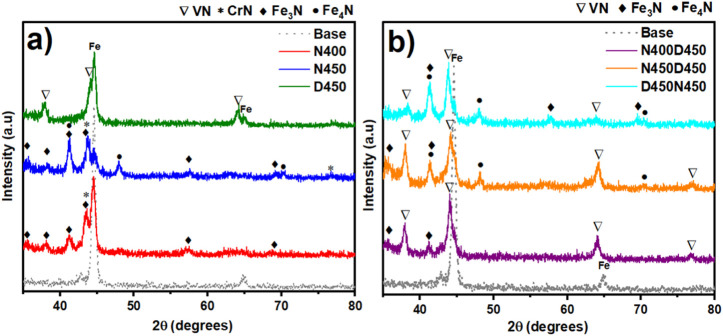
X-ray diffraction patterns of the base sample and the samples subjected to (a) single and (b) duplex treatments, highlighting the Fe_3_N, Fe_4_N, CrN, and VN phases.

Sample D450, produced by CCPD, exhibits an iron phase and a secondary vanadium nitride (VN) phase. Despite the presence of VN in the diffractogram, the higher intensity of the iron peak (2θ = 44.7°) suggests the formation of a thin VN surface film. According to Tahchieva et al. (2019), films composed of VN exhibit high hardness and good corrosion resistance.^39^ Thus, the combination of the modification promoted by the CCPN process, which consists of the formation of iron and chromium nitrides on the substrate, with the deposition of a thin vanadium nitride film, can enhance the mechanical strength of AISI 409 steel by creating a hardness gradient toward the surface.

The samples subjected to duplex treatment presented the resulting phases of CCPN and CCPD treatments, respectively. However, a slight increase in the intensity of the peaks related to the VN phase can be noted. This may indicate an increase in the thickness of the film as a function of the CCPD temperature. The Fe phase appears less intense in the diffractograms of Figure 2b due to the vanadium nitride film above the nitrided layer. Sample D450N450 showed a reduction in VN peaks and a greater intensity of nitride precipitates from the nitriding process. This may impair the behavior of the coating when subjected to corrosive environments, despite the formation of chromium nitrides not being observed. Therefore, the XRD patterns of the treated samples confirm the formation of iron and vanadium nitrides due to plasma processing. SEM and EDS analyses were carried out to investigate the morphology and percentage elemental content of the modified surfaces (Figure 3).

**Figure 3:**
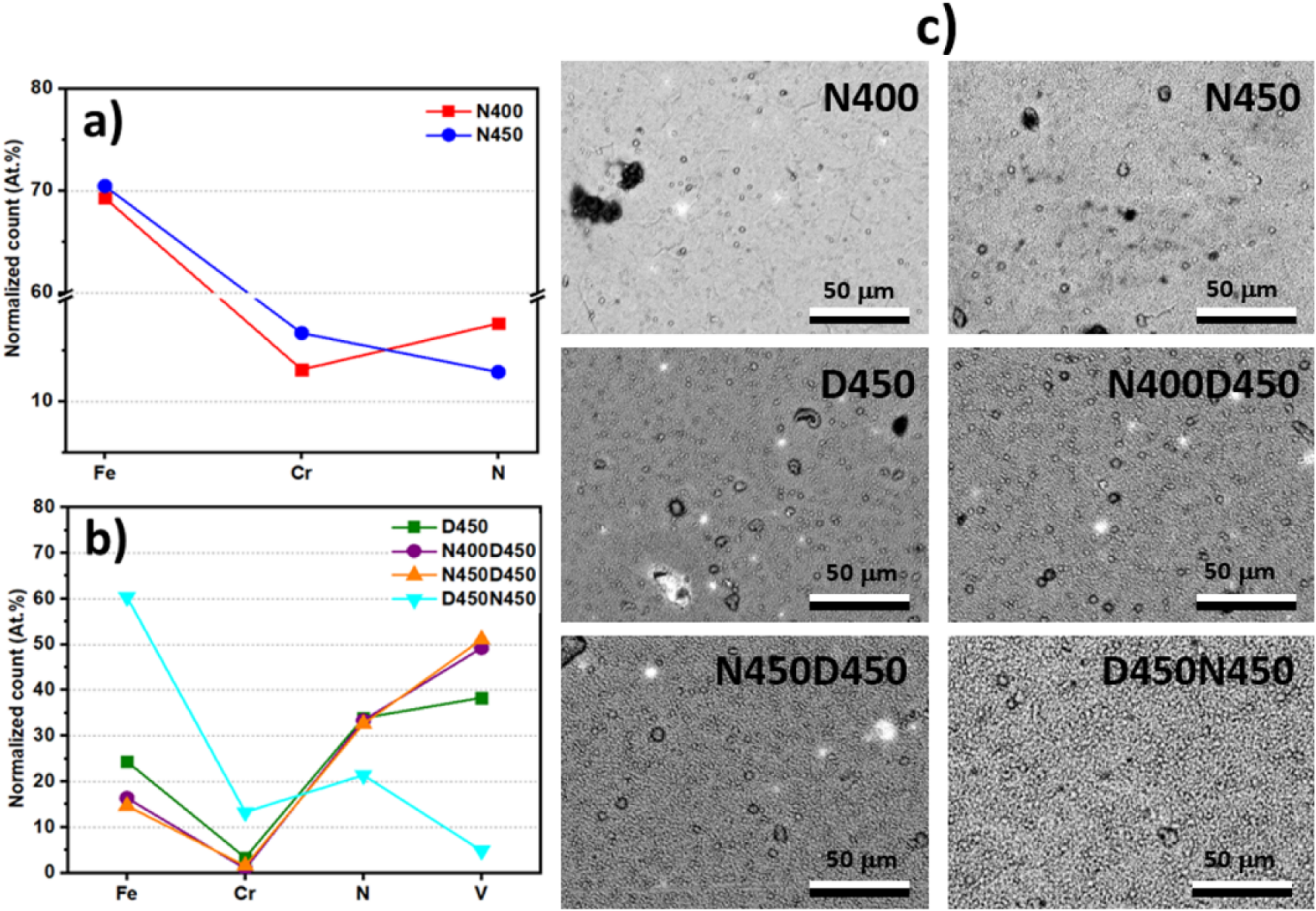
SEM images and EDS atomic percentages for samples after (a) single and (b) duplex treatments, highlighting surface composition changes in Fe, Cr, N, and V. Surface areas where elemental contents were measured by EDS (c).

Figure 3 shows each treated sample’s arithmetic mean of the SEM/EDS results. Figure 3a shows that increasing the nitriding temperature from 400 to 450 °C caused a slight increase in the chromium content present in the AISI 409 steel coating. This increase is associated with the formation of the chromium nitride phase, which is more evident in the diffractogram of sample N450 (Figure 2a). Figure 3b compares the normalized atomic percentage of Fe, Cr, N, and V elements in the samples submitted for deposition and duplex treatment. The sample submitted only to the VN deposition process (D450) showed 38% vanadium and 34% nitrogen, corroborating the XRD results, which showed the VN phase. This composition indicates the incorporation of vanadium and nitrogen into the surface layer, typical for treatments aimed at enhancing surface hardness and wear resistance by forming hard vanadium nitrides.

The N400D450 and N450D450 samples, subjected to duplex treatment, showed an increase in vanadium content and a reduction in iron content compared to the D450 sample. This behavior can be attributed to the more efficient deposition of the VN film, which forms a continuous and protective surface layer. EDS analyses were performed at an acceleration voltage of 15 kV for all samples. Under these conditions, the lower intensity of the iron signal in the duplex-treated samples (CCPN–CCPD) may be related to the greater thickness and density of the VN layer, which limits the penetration of primary electrons or attenuates the emission of iron-characteristic X-rays originating from deeper regions. Even under identical analysis conditions, the composition and thickness of the outer layer influence the excitation volume and, consequently, the EDS spectral response. The reduction in iron content may also indicate a denser and less porous nitrided layer, further hindering electron penetration into the substrate. This means that nitriding treatment before deposition favors the growth of the protective vanadium nitride phase.

The results show no significant change in the surface atomic composition of the films produced on the N400D450 and N450D450 samples. This is not observed in the structural results of the diffractograms, as there is an increase in nitride precipitates with increasing nitriding temperature (Figure 2b).

Figure 3b shows that reversing the order of the processes in the duplex treatment significantly affects the qualitative atomic content. The D450N450 sample shows a substantial presence of iron, indicating that the deposition process was less effective in incorporating vanadium into the surface and may have promoted defects in the film formation, allowing the detection of X-rays characteristic of iron from the nitrided layer. Furthermore, subsequent nitriding at 450 °C did not increase the nitrogen content effectively. The increased chromium content suggests the formation of chromium nitrides during the nitriding stage postdeposition. The vanadium content dropped from 50% to 5%, indicating that this approach hinders the formation of an efficient VN layer on AISI 409. Figure 3c shows that there was no significant change in surface morphology between samples N400 and N450. On the other hand, samples subjected to the CCPD process (D450, N400D450, and N450D450) showed a slight increase in grain size. The D450N450 sample presented larger grains with more evident contours. This may justify low densification in the formation of the layer and the consequent detection of elements in more internal regions. Having established the surface composition, cross-sectional SEM was performed to determine nitride layer thickness and interface integrity (Figure 4).

**Figure 4:**
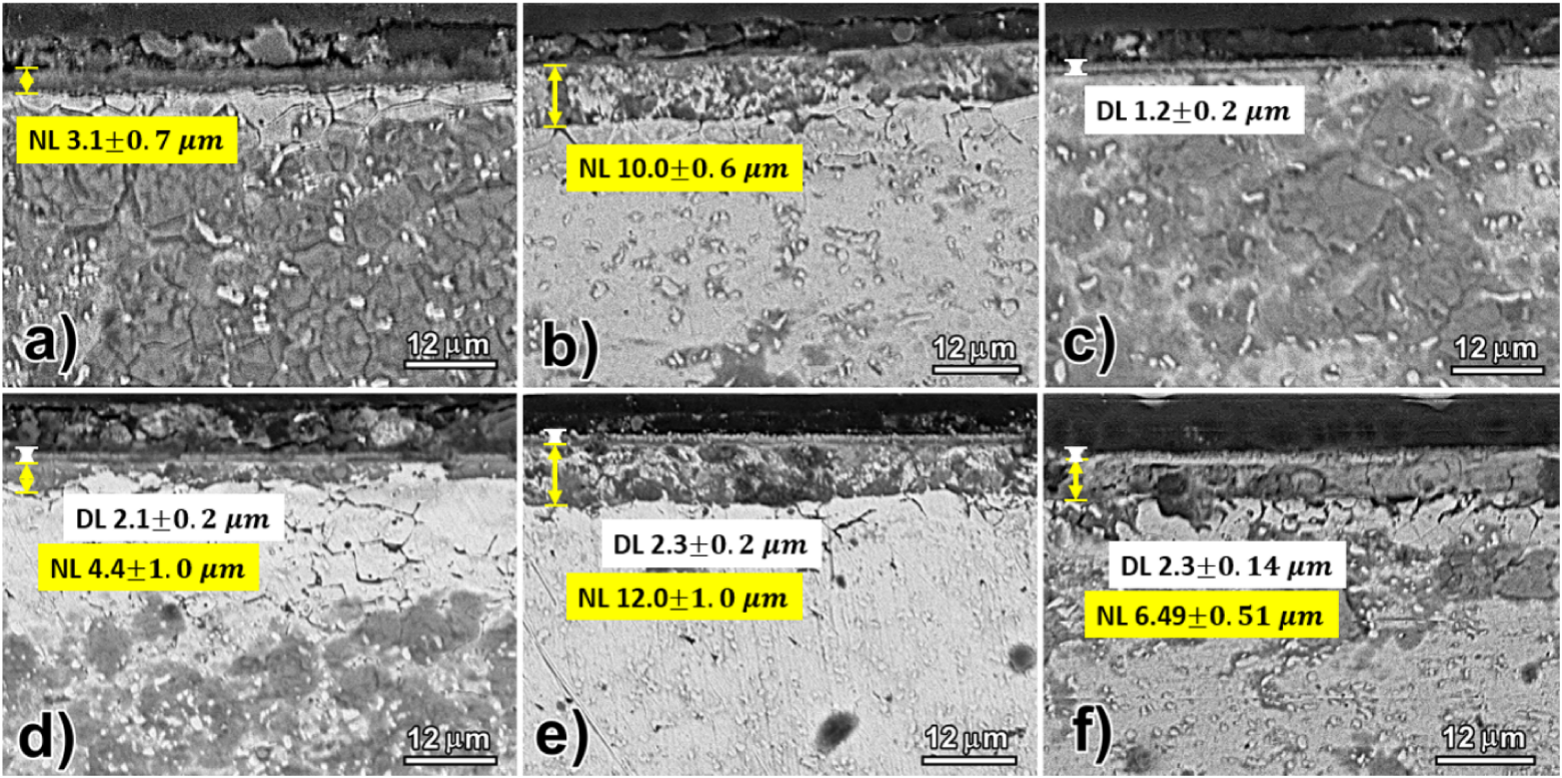
Cross-sectional SEM images highlighting the nitrided layers and VN films. Samples: (a) N400, (b) N450, (c) D450, (d) N400D450, (e) N450D450, (f) D450N450.

Figure 4 displays the formation of thick, considerably uniform nitrided layers (NL), demonstrating a direct relationship between layer thickness and treatment temperature. Specifically, a nitrided layer of 3.1 μm was observed in sample N400, while sample N450 exhibited a thickness of 10.0 μm. Previous studies have identified the formation of nitrides in darker regions of the nitrided layer, attributed to the high concentration of chromium in the substrate and the intense binding energy between chromium and nitrogen.^40,41^

The cross-section SEM analysis reveals that the vanadium cathodic cage plasma deposition (CCPD) process effectively creates uniform films with thicknesses ranging from 1.2 to 2.3 μm across treated samples. The single deposition process (D450) produces the smallest deposited layer (DL) (1.2 μm), while duplex treatments achieve a more significant layer between 2.1 and 2.2 μm. This phenomenon is explained by prenitriding treatments creating a hardened and roughened surface that enhances the adhesion and deposition efficiency of the subsequent vanadium layer. The nitrided layer is a robust foundation that improves the interaction between the substrate and the deposited material, facilitating the formation of a thicker coating. The increase in layer thickness in duplex treatments is also observed in other works,^42–44^ suggesting that forming a thick, hardened layer is the main mechanism for mechanical support for the subsequently deposited ceramic coating.

It is also observed that the duplex treatments conducted at 450 °C (N450D450 and D450N450) produced slightly thicker layers than the duplex treatment at a lower temperature in the nitriding phase (N400D450). This increase in thickness is likely due to the higher temperature promoting a more extensive diffusion of nitrogen and vanadium into the substrate, enhancing the overall deposition process. Higher temperatures facilitate greater atomic mobility, leading to a more pronounced and uniform layer growth. The elevated temperature may also promote the formation of more reactive sites on the substrate surface, contributing to the increased layer thickness observed in the higher-temperature treatments. The notable thickness observed, particularly in the N450D450 sample, is attributed to the synergistic effects of prenitriding treatments, which enhance subsequent vanadium deposition. Variations in processing parameters such as temperature and duration also contribute to the differences in deposition thickness, with higher temperatures and longer durations promoting thicker coatings.

Figure 4f illustrates the D450N450 treatment, characterized as an inverse duplex process, which resulted in a thinner nitrided layer compared to other duplex treatments. This outcome is primarily due to the properties of AISI 409 steel, a ferritic stainless steel rich in alloying elements like chromium. In the initial vanadium deposition phase of the inverse duplex treatment, the smooth surface generated is less conducive to effective nitriding. As a result, the subsequent nitriding at 450 °C is less efficient, leading to lower nitrogen content and a reduced nitrided layer thickness compared to other treatment sequences. Moreover, nitrogen diffusion in VN occurs via migration through octahedral nitrogen vacancies, a mechanism commonly observed in nitride materials. The activation energy for nitrogen diffusion in VN, approximately 2.92 eV, is significantly higher than that in iron nitride, which is around 0.95 eV. This suggests that the diffusion process in VN may be slower when passing through the previously deposited film.^45,46^

The high chromium content in AISI 409 steel facilitates the formation of stable chromium nitrides during nitriding. These nitrides have a strong binding energy with nitrogen, reducing the availability of reactive sites for vanadium nitride deposition. Consequently, the D450N450 sample exhibited lower vanadium percentages and less effective VN layer formation, as evidenced by the significant presence of iron in this sample, indicating that the deposition process was less effective in saturating the surface with vanadium. These observations highlight the importance of treatment sequence, with nitriding followed by deposition proving more effective in forming thick, uniform surface layers than the reverse sequence.^22^ With the layer dimensions defined, we evaluated their mechanical reinforcement via Vickers microhardness profiles (Figure 5).

**Figure 5:**
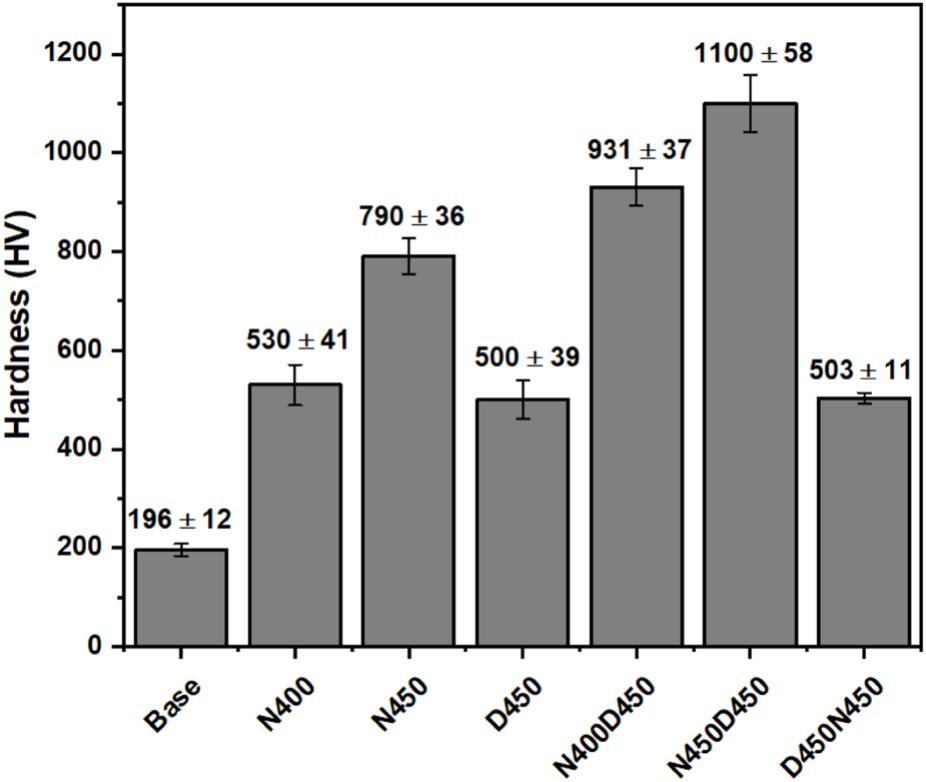
Surface Vickers hardness of the analyzed samples, with corresponding standard deviations shown by the error bars.

Figure 5 presents the average Vickers hardness of untreated and plasma-treated AISI 409 surfaces. The base material exhibited a hardness of 196 HV, while all plasma treatments produced significant hardening. SEM micrographs confirmed the formation of uniform nitride layers, and XRD analysis identified Fe_3_N, Fe_4_N, and CrN phases whose intrinsic high hardness directly correlates with the elevated microhardness values.^43,47^

Single-stage cathodic cage plasma nitriding at 400 °C (N400) raised surface hardness to 530 HV, and raising the temperature to 450 °C (N450) further increased hardness to 790 HV, corresponding to improvements of 170% and 300% over the untreated substrate. As shown in Figure 4a,b, these samples develop a composite layer on the ferritic matrix and a much thicker diffuse nitride zone that grows with temperature.^43,44,48^

Sample D450, deposited with vanadium using the cathodic cage deposition technique, had the thinnest layer thickness, as shown in Figure 4c. However, despite this characteristic, the thin film deposited shows high hardness, as can be seen in Figure 5. The hardness values found were similar to those of the N400 sample, but this was due to the depth of penetration of the Vickers indentation, which exceeded 10% of the VN film thickness.

Vanadium deposition alone at 450 °C (D450) produced the thinnest coating (Figure 4c) but achieved a hardness comparable to the N400 sample. Because the Vickers indentations penetrated more than 10 % of the VN film thickness, the underlying substrate’s elastoplastic response significantly influences the measured values. Thus, the true hardness of the VN layer is likely higher than recorded, and any detached VN particles could act as abrasives during tribological contact.

Duplex sequences yielded the highest hardness: N400D450 achieved 931 HV, while N450D450 peaked at 1100 HV. By contrast, the inverse sequence (D450N450) produced hardness values comparable to single-stage treatments, underscoring the importance of prenitriding. The initial formation of a thick, hard layer provides mechanical support for a thinner deposited ceramic coating, resulting in a surface with good tribological properties and corrosion resistance.^42^ In AISI 409, a ferritic stainless steel already alloyed with chromium, titanium, and other elements, adding more metallic species via deposition can impede nitrogen diffusion in a follow-up nitriding step. The deposited VN layer acts as a diffusion barrier, limiting nitrogen uptake and hindering nitride network growth. Consequently, nitriding after deposition cannot establish the robust subsurface nitride structure achieved when nitriding precedes deposition, making the inverse duplex approach less effective for stainless steels.^22^

To place these results in context, the N450D450 duplex treatment delivers a substantial improvement over previous plasma-based methods on AISI 409. Berton et al. (2017), for example, reported ≈500 HV at 200 μm depth after single-step plasma nitriding and solution-heat treatment; our duplex route more than doubles this value.^49,50^ Moreover, our hardness level is comparable to the ∼1000 HV achieved on AISI 420 martensitic stainless steel at 450 °C,^50^ despite AISI 409’s lower alloy content. Unlike high-temperature nitriding (>500 °C), which can exceed 1200 HV at the expense of corrosion resistance,^51^ our duplex CCPN → CCPD treatment operates at 400–450  °C and maintains the corrosion stability of ferritic stainless steel while achieving martensitic-grade hardness.

Figure 6 shows the average values of the hardness profiles of the samples as a function of depth. The graph shows the maximum limit of the layer(s) resulting from the treatments, as seen in Figure 4. The limit of the layer(s) is located by the intersection between the hardness profile curve and the red dashed horizontal line which represents the layer depth criterion proposed by the DIN 50190-3 NHT standard which describes the limit depth of the layer in the zone where the hardness of the profile reaches a value of 50 HV above the hardness of the substrate (196.7 HV + 50 HV = 246.7 HV).^52,53^

**Figure 6:**
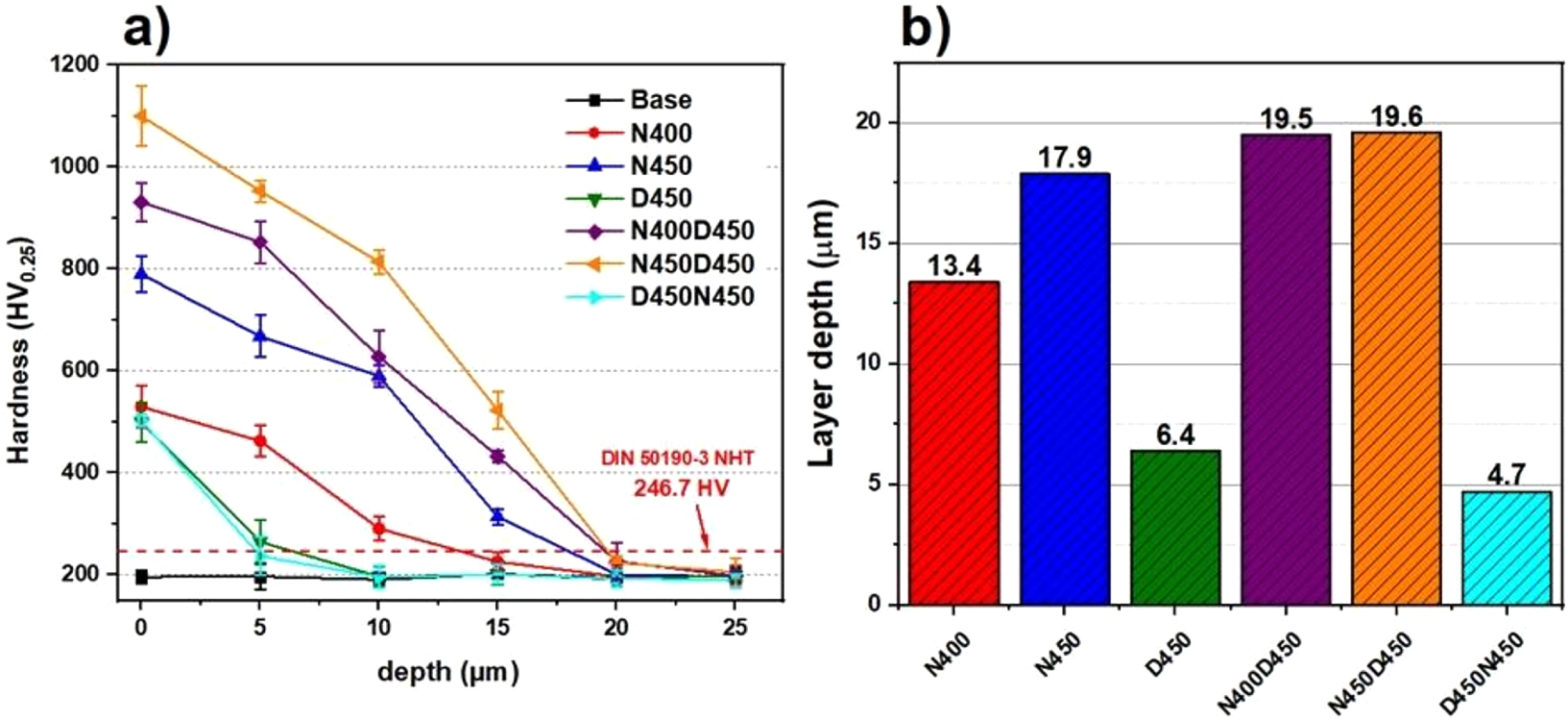
(a) Vickers hardness profiles as a function of depth and (b) total thickness of the treated layers, estimated according to the DIN 50190-3 NHT standard.

As previous studies^54–56^ supported, hardness values gradually decrease along the depth profile as measurements are taken inside the sample. A sharper drop is observed when the identifications are made close to the boundary of the thermochemical treatment layer(s), persisting until it stabilizes at values close to those of the untreated sample, which occurred at around 20 μm depth for the samples subjected to the duplex treatment (CCPN followed by CCPD). This analysis is essential to confirm the films’ approximate length and hardness variation throughout the layer. It is worth noting that the N400D450 and N450D450 duplex treatments, as well as the N450 treatment, showed a smoother decrease along the depth, indicating the ability of these surfaces to more effectively resist plastic deformation and the effects resulting from sliding contact.

The hardness data obtained highlight the surface processes’ effectiveness compared to the untreated sample. For example, the highest average hardness reached 1100 HV, while the original sample had 196 HV, reflecting a percentage increase of 461.22% compared to the untreated state.

The hardness data obtained highlight the surface processes’ effectiveness compared to the untreated sample. For example, the highest average hardness reached 1100 HV, while the original sample had 196 HV, reflecting a percentage increase of 461.22% compared to the untreated state. To link these hardness gains to functional performance, ball-on-disc wear tests were conducted under identical conditions (Figure 7).

**Figure 7:**
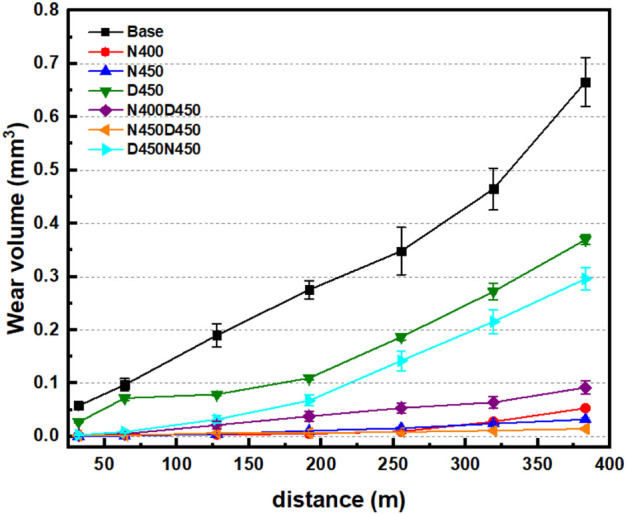
Wear volume as a function of sliding distance in the ball-on-disc tribological test for the base sample and samples subjected to nitriding, deposition, and duplex treatments.

Figure 7 shows the behavior of the AISI 409 samples subjected to the sliding contact of a ball under the action of a force of 8 N. The results showed that all the plasma-treated samples had improved wear resistance compared to the base sample (untreated), and the wear volume vs distance measurements supported this. The samples subjected to the simple nitriding treatment (N400 and N450) showed similar behavior, with a very low volume of material lost at the end of the test. However, the N450 sample proved slightly more resistant than the N400 sample in the final cycles. Therefore, the excellent tribological behavior of these samples is due to the formation of the iron nitride (Fe_3_N and Fe_4_N) and chromium nitride (CrN) phases, shown in Figure 2a, which provide good surface mechanical resistance. In addition, the better relative performance of the N450 sample can also be explained by the greater depth of the layer modified by the treatment, as seen in Figure 4a,b.

The surface of sample D450 showed the worst tribological behavior among the treated samples. The simple CCPD deposition treatment with a vanadium cage produced a thin film of approximately 1.2 μm without a hardness gradient toward the surface, as is common in nitriding or duplex treatments consisting of nitriding followed by deposition. This absence of a hardness gradient, as shown in Figure 6a, makes the hard VN film more fragile and less anchored to the steel surface. As a result, the VN film becomes more susceptible to degradation during tribological contact. The results in Figure 7 show that the D450 sample has a slightly higher wear rate (slope of the curve) than the base sample in the first test cycles. This can be explained by the probable easy detachment of the hard film in the form of particulates that initially behave like abrasives. After the reduction of this sliding interaction mediated by hard particles, the contact becomes more uniform, smoothing out the tribological behavior. For this reason, in the intermediate cycles, there is a zone of lower wear rate (between 80 and 200 m). Finally, between 200 and 370 m, there is a similar wear behavior to that observed in the base sample (same slope), meaning that, in this phase, contact occurs between the ball and the AISI 409 substrate, which has not been modified by the treatment.^57,58^

The samples subjected to the duplex treatment (CCPN followed by CCPD) also showed a low wear rate during the tribological test when compared to the base, D450, and D450N450 samples. The N400D450 sample, for example, achieved 76% less wear than the D450 sample. However, it performed less well than the nitrided-only samples (N400 and N450). On the other hand, sample N450D450 showed the best tribological performance of all the samples analyzed. The insignificant amount of wear at the end of the analysis can be explained by the combination of high surface hardness and greater layer depth modified by the duplex treatment (see Figures 4e and 5). The fact that samples N450 and N450D450 showed the best wear resistance results indicates that prior nitriding at 450 °C is a relevant factor for the final response of the coating. On the other hand, sample D450N450, subjected to the reverse duplex treatment, showed the worst tribological performance among the duplex treatments. This is due to the reduced mechanical strength measured by the surface microhardness test, the hardness profile characteristic of a shallow surface modification (similar to that observed in the D450 sample) and the apparent lower densification of the VN film which can be deduced from the EDS results which show a high Fe content and low Cr, N and V contents, indicating greater penetration of the accelerated electron beam due to possible malformation of the VN film.^59^

During the wear tests, the friction coefficient of the sample with the 52100 steel sphere was monitored, as shown in Figure 8. Both the treated samples and the untreated AISI 409 stainless steel exhibited a uniform friction coefficient throughout the wear test, primarily due to the consistent formation of wear-resistant phases on the surface, as shown in XRD and wear volume results. All samples demonstrated consistency in their friction coefficient values, with no significant variations to create peaks or valleys in the graph, except for the N400 sample, which showed an increase in the friction coefficient after 1000 s. This increase is likely due to the breakdown of the initial protective nitride layer, leading to increased surface roughness and higher friction.^60^

**Figure 8:**
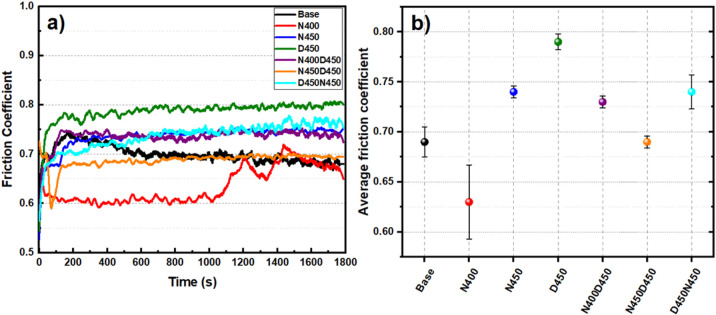
(a) Friction coefficient of AISI 409 steel with and without treatment(s) and (b) average friction coefficient calculated from 200 s to the end of the test.

The base sample showed an increase in the coefficient of friction in the initial moments of the tribological test (0 to 200 s). This increase can be explained by the slight surface hardening resulting from the sample preparation process described in the methodology.^61^ After the debris is pulled away from this harder area, the contact between the surfaces becomes softer, and consequently, the friction coefficient values start to decrease.^62^ On the other hand, the other samples did not show this behavior in the initial phase. The N400 and N450D450 samples had lower average friction coefficient values than the base sample. However, the contact between the N400 sample and the ball was unstable from 1000 s onward, reflecting the increase in the standard deviation of the average friction coefficient result (see Figure 8b). It can be seen that the base, N400, and D450N450 samples had larger measurement deviations than the other samples, which had stable and constant tribological behavior. In the base sample, this deviation was due to initial hardening, in the N400 sample, due to a disturbance in the final phase of the test (possibly the ball reaching the hardening region), and in the D450N450 sample, there was a continuous increase in the coefficient of friction, indicating the presence of a harder layer, with low anchorage and possibly rougher as a result of the reverse duplex treatment. In addition to mechanical durability, corrosion resistance is a critical requirement for components exposed to aggressive environments; thus, electrochemical tests were performed to evaluate the protective behavior of the treated surfaces (Figure 9).

**Figure 9:**
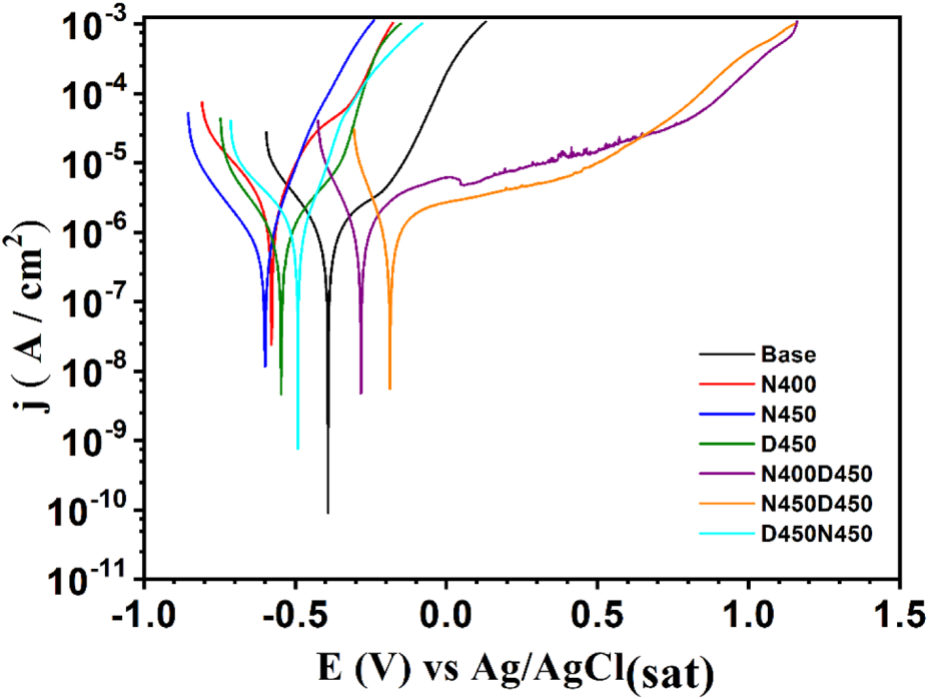
Potentiodynamic polarization curves in 3.5% NaCl solution, showing a positive shift in corrosion potential for the duplex treatments (CCPN followed by CCPD).

Figure 9 presents the potentiodynamic polarization curves of the treated samples compared to the untreated base material, with the parameters extracted by Tafel extrapolation summarized in Table 2. The results reveal significant variations in the corrosion potential (*E*_corr_) and the corrosion current density (*j*_corr_), both indicative of the corrosion behavior. The N400 and N450 samples, subjected exclusively to nitriding, exhibited distinct responses relative to the base sample. Sample N400 showed an *E*_corr_ = −575 mV and *j*_corr_ = 1.98  μA/cm^2^, indicating poor corrosion resistance due to both early corrosion onset and a high rate of corrosion progression. The cathodic slope (β_c_) and anodic slope (β_a_) values obtained from Tafel’s linear extrapolations also indicate the formation of a layer that is not very protective against the reduction reaction in the cathodic region and the oxidation of the metal.

**Table 2:** Parameters Extracted from the Potentiodynamic Polarization Curves from Tafel Extrapolation

Sample	β_c_ (mV/dec)	β_a_ (mV/dec)	*E*_corr_ (mV)	*j*_corr_ (μA/cm^2^)
Base	166.65	243.40	–396	0.80
N400	183.11	117.00	–575	1.98
N450	167.22	86.32	–605	0.50
D450	155.83	181.83	–552	0.73
N400D450	105.15	546.11	–319	1.72
N450D450	83.02	1013.17	–221	1.59
D450N450	205.98	83.85	–476	0.97

In contrast, sample N450 exhibited the most negative *E*_corr_ (−605 mV), suggesting a high susceptibility to corrosion initiation. However, its *j*_corr_ = 0.50 μA/cm^2^ was the lowest in the data set, indicating a slower corrosion rate under steady-state conditions. This difference can be attributed to the formation of denser and more protective nitride layers at higher nitriding temperatures. Additionally, the presence of the CrN phase identified by X-ray diffraction (XRD) in both samples further contributes to reduced corrosion resistance, as the formation of this compound consumes chromium from the matrix, thereby compromising the passive chromium oxide layer that naturally protects stainless steel.^63^

Duplex-treated samples N400D450 and N450D450, processed via nitriding followed by vanadium nitride (VN) deposition, demonstrated the best overall corrosion performance. Sample N400D450 achieved an *E*_corr_ = −319 mV and *j*_corr_ = 1.72 μA/cm^2^, while N450D450 reached *E*_corr_ = −221 mV and *j*_corr_ = 1.59 μA/cm^2^. Despite the high *j*_corr_ values compared to the base sample, these two samples showed more positive corrosion potentials, indicating less susceptibility to the onset of the corrosive process, as well as good anodic protection recorded by the high β_a_ values, 546.11 and 1013.17 mV/dec, respectively. These results reflect the synergistic effects of the two processes, promoting the formation of a hardened surface layer with good adhesion and mechanical support, which reduces the ingress of aggressive species and favors electrochemical stability.

Sample D450, subjected exclusively to VN deposition, exhibited a corrosion potential of −552 mV and a corrosion current density of 0.73 μA/cm^2^, values that indicate moderate protection in a 3.5% NaCl environment. The Tafel parameters extracted, β_c_ = 155.83 mV/dec and β_a_ = 181.83 mV/dec, reveal that both the reduction and oxidation reactions are partially controlled by the formed layer. This relatively balanced electrochemical response suggests that the VN film acts as a limited barrier, reducing the corrosion rate compared to the base sample, but without providing protection as effective as that observed in the duplex treatments.

The D450N450 sample, which underwent reverse duplex treatment (deposition followed by nitriding), exhibited *E*_corr_ = −476 mV, more negative than that of the untreated base sample (−396 mV), and a *j*_corr_ = 0.97 μA/cm^2^, indicating inferior corrosion resistance compared to the untreated sample. This behavior may be attributed to the inadequate sequencing of the processes, which hindered the efficient formation of protective layers and resulted in an unfavorable distribution of nitrogen and vanadium.^64–66^ This is further evidenced by the XRD patterns (Figure 2), which showed a higher relative intensity of Fe_3_N and Fe_4_N phases at the expense of VN formation, while the EDS analysis (Figure 3) revealed lower vanadium and nitrogen contents in the D450N450 sample compared to the other duplex-treated conditions. Additionally, the surface image of the D450N450 sample obtained by SEM (Figure 3b) shows more distinct and well-defined grain boundaries, which may indicate lower density and compaction of the vanadium nitride (VN) layer formed. This morphology favors the exposure of the underlying microstructure to the electrolyte, compromising the stability and effectiveness of the protective coating in the corrosive environment.

In summary, the combined evaluation of *E*_corr_ and *j*_corr_ confirms that the conventional duplex treatments (N400D450 and N450D450) are the most effective for corrosion protection of AISI 409 steel in a 3.5% NaCl environment. The integration of nitriding and VN deposition results in a more stable, dense, and adherent coating that delays the onset of corrosion and reduces its progression rate. Although the cathodic Tafel slopes (β_c_) of these samples were lower (105.15 and 83.02 mV/dec, respectively), suggesting that the oxygen reduction reaction may still occur to some extent, anodic control predominates in the corrosion mechanism of ferritic stainless steels, making β_a_ the most decisive factor. In this context, duplex coatings mitigate the main vulnerability of AISI 409 (susceptibility to anodic dissolution) by forming compact and chemically stable barrier layers.

## 4 Conclusions

In this work, cathodic cage plasma nitriding (CCPN), vanadium nitride deposition (CCPD), and their sequential duplex combinations were applied to AISI 409 ferritic stainless steel to enhance hardness, wear resistance, and corrosion behavior. Single-stage CCPN at 400 and 450 °C produced uniform Fe_3_N/Fe_4_N layers that increased surface hardness from 196 HV to 530 HV and 790 HV, respectively, and significantly reduced wear volume. CCPD alone deposited a thin VN film with comparable hardness to N400 but lacked a graded interlayer, which limited its tribological performance under sliding contact.

Duplex treatments combining CCPN followed by CCPD yielded the most substantial gains. The N400D450 treatment reached 931 HV and the N450D450 peaked at 1100 HV, which is greater than the hardness reported for single-step plasma-nitrided AISI 409. Wear tests demonstrated up to an 97% reduction in wear volume versus the untreated substrate, confirming the synergistic effect of a hard interlayer supporting a wear-resistant VN cap.

In terms of electrochemical stability in a corrosive environment, the presence of the VN film contributed to reducing the material’s tendency toward spontaneous corrosion. This effect is supported by the more noble *E*_corr_ values observed in the duplex-treated samples (CCPN + CCPD) when compared to the untreated sample. Moreover, the high anodic Tafel slope observed for the N450D450 sample (β_a_ = 1013.17 mV/dec) indicates the formation of a highly stable passive layer, with low susceptibility to metal dissolution processes, an essential characteristic for ferritic stainless steels, which inherently exhibit lower passivation stability due to their reduced nickel content.

These improvements significantly enhance the application potential of AISI 409 stainless steel in more aggressive industrial environments, enabling its use in components that require both mechanical strength and corrosion resistance, without compromising the material’s economic viability.
